# Supplementary material for: The Online Processing of Korean Case by Native Korean Speakers and Second Language Learners as Revealed by Eye Movements
Source: Brain Sci. 2022 Sep 11;12(9):1230. doi: 10.3390/brainsci12091230 (PMC9496816; doi:10.3390/brainsci12091230)
Supplement: Supplementary file 1 [file brainsci-12-01230-s001.zip › brainsci-1873311-supplementary.pdf]

Table S1. Accusative monotransitive (1_20) and dative experimental matierials (21 -40), in canonical (a) and scrambled (b) word order. Materials were presented orally in Experiment 1 and orthographically in Experiment 2. The probe questions were only presented in Experiment 2.

Canonical SOVword order	Scrambled OSVword order	Probe question (Experiment 2)	Correct response
1a 소녀가 요리사를 민다<br>sonyeoka yorisareul minda<br>girl(nom) chef(acc) push(pres ind)	1b 요리사를 소녀가 민다<br>yorisareul sonyeoka minda<br>chef(acc) girl (nom) push(pres ind)	1. 소녀는 미나요?<br>Sonyaneun minayo?<br>Girl(top) push(pres ind)	**Y**
2a 어른이 아이를 민다<br>eoreuni aireul minda<br>adult(nom) child(acc) push(pres ind)	2b 아이를 어른이 민다<br>aireul eoreuni minda<br>child(acc) adult(nom) push(pres ind)	2. 아이는 미나요?<br>aineun minayo?<br>Child(top) push(pres ind)	N
3a 남자가 여자를 민다<br>namjaka yeojareul minda<br>man(nom) woman(acc) push(pres ind)	3b 여자를 남자가 민다<br>yeojareul namjaka minda<br>woman(acc) man(nom) push(pres ind)	3 ; 남자는 미나요?<br>namjaneun minayo ?<br>Man(top) push(pres ind)	Y
4a 학생이 선생님을 민다<br>haksaengi seonsaengnimeul minda<br>student(nom) teacher(acc) push(pres ind)	4b 선생님을 학생이 민다<br>seonsaengnimeul haksaengi minda<br>teacher(acc) student(nom) push(pres ind)	4. 선생님은 미나요?<br>Seonsaengnimeun minayo ?<br>Teacher(top) push(pres ind)	N
5a 소년이 소녀를 본다<br>sonyeoni sonyeoreul bonda<br>boy(nom) girl(acc) see(pres ind)	5b 소녀를 소년이 본다<br>sonyeoreul sonyeoni bonda<br>girl(acc) boy(nom) see(pres ind)	5. 소년은 보나요?<br>seonyeoneun bonayo?<br>boy(top) see(pres ind	Y
6a 환자가 간호사를 본다<br>hwanjaka kanhosareul bonda<br>patient(nom) nurse(acc) see(pres ind)	6b 간호사를 환자가 본다<br>kanhosareul hwanjaka bonda<br>nurse(acc) patient(nom) see(pres ind)	6. 환자는 보나요?<br>hwanjaneun bonnayo ?<br>patient(top) see(pres ind)	Y
7a 선생님이 학생을 본다<br>seonsaengnimi haksaengeul bonda<br>teacher(nom) student(acc) see(pres ind)	7b 학생을 선생님이 본다<br>haksaengeul seonsaengnimi bonda<br>student(acc) teacher(nom) see(pres ind)	7. 학생은 보나요?<br>haksaengeun bonayo ?<br>student(top) see(pres ind)	N
8a 요리사가 여자를 본다<br>yorisaka yeojareul bonda<br>chef(nom) woman(acc) see(pres ind)	8a 여자를 요리사가 본다<br>yeojareul yorisaka bonda<br>woman(acc) chef(nom) see(pres ind)	8. 여자는 보나요?<br>yeojaneun bonayo ?<br>woman(top) see(pres ind)	N
9a 아이가 개를 사랑한다<br>aika kaereul saranghanda<br>child(nom) dog(acc) love(pres ind)	9b 개를 아이가 사랑한다<br>kaereul aika saranghanda<br>dog(acc) child(nom) love(pres ind)	9. 개는 사랑하나요?<br>kaeneun saranghanayo ?<br>dog(top) love(pres ind)	N
10a 의사가 간호사를 사랑한다<br>euisaka kanhosareul saranghanda<br>doctor(nom) nurse(acc) love(pres ind)	10b 간호사를 의사가 사랑한다<br>kanhosareul euisaka saranghanda<br>nurse(acc) doctor(nom) love(pres ind)	10. 의사는 사랑하나요?<br>euisaneun saranghanayo ?<br>doctor(top) love(pres ind)	Y
11a 여자가 남자를 사랑한다<br>yeojaka namjareul saranghanda	11a 남자를 여자가 사랑한다<br>namjareul yeojaka saranghanda	11. 남자는 사랑하나요?<br>namjaneun(top) love(pres ind)	N

woman(nom) man(acc) love(pres ind)	man(acc) woman(nom) love(pres ind)	man(top) love(pres ind)	
12a 소녀가 소년을 사랑한다<br>sonyeoka sonyeoneul saranghanda<br>girl(nom) boy(acc) love(pres ind)	12b 소년을 소녀가 사랑한다<br>sonyeoneul sonyeoka saranghanda<br>boy(acc) girl(nom) love(pres ind)	12 소녀는 사랑하나요?<br>sonyeoneun love(pres ind)<br>girl(top) love(pres ind)	Y
13a 아이가 어른을 깨운다<br>aika eoreuneul kkaeunda<br>child(nom) adult(acc) wake up (pres ind)	13b 어른을 아이가 깨운다<br>eoreuneul aika kkaeunda<br>adult(acc) child(nom) wake up (pres ind)	13. 아이는 깨우나요?<br>aineun kkaeunayo?<br>child(top) wake up(pres ind)	Y
14a 소년이 남자를 깨운다<br>sonyeoni namjareul kkaeunda<br>boy(nom) man(acc) wak up (pres ind)	14b 남자를 소년이 깨운다<br>namjareul sonyeoni kkaeunda<br>man(acc) boy(nom) wak up (pres ind)	14. 소년은 깨우나요?<br>sonyeoneun kkaeunayo?<br>boy(top) wake up(pres ind)	Y
15a 간호사가 의사를 깨운다<br>kanhosaka euisareul kkaeunda<br>nurse(nom) doctor(acc) wake up (pres ind)	15b 의사를 간호사가 깨운다<br>euisareul kanhosaka kkaeunda<br>doctor(acc) nurse(nom) wake up (pres ind)	15. 의사는 깨우나요?<br>euisaneun kkaeunayo?<br>doctor(top) wake up(pres ind)	N
16a. 여자가 요리사를 깨운다<br>yeojaka yorisareul kkaeunda<br>woman(nom) chef(acc) wake up (pres ind)	16b. 요리사를 여자가 깨운다<br>yorisareul yeojaka kkaeunda<br>chef(acc) woman(nom) wake up (pres ind	16. 요리사는 깨우나요?<br>yorisaneun kkaeunayo?<br>chef(top) wake up	N
17a. 요리사가 소녀를 쫓는다<br>yorisaka sonyeoreul jjochneunda<br>chef(nom) girl(acc) chase(pres ind)	17b. 소녀를 요리사가 쫓는다<br>sonyeoreul yorisaka jjochneunda<br>girl(acc) chef(nom) chase(pres ind)	17. 요리사는 쫓나요?<br>yorisaneun jjochnayo ?<br>chef(top) chase(pres ind)	Y
18a. 개가 아이를 쫓는다<br>kaeka aireul jjochneunda<br>dog(nom) child(acc) chase(pres ind)	18b. 아이를 개가 쫓는다<br>aireul kaeka jjochneunda<br>child(acc) dog(nom) chase(pres ind)	18. 아이는 쫓나요?<br>aineun jjochnayo?<br>child(top) chase	N
19a. 남자가 소년을 쫓는다<br>namjaka sonyeoneul jjochneunda<br>man(nom) boy(acc) chase(pres ind)	19b. 소년을 남자가 쫓는다<br>sonyeoneul namjaka jjochneunda<br>boy(acc) man(nom) chase(pres ind)	19. 남자는 쫓나요 ?<br>namjaneun jjochnayo?<br>man(top) chase(pres ind)	Y
20a. 간호사가 환자를 쫓는다<br>kanhosaka hwanjareul jjochneunda<br>nurse(nom) patient(acc) chase(pres ind)	20b. 환자를 간호사가 쫓는다<br>hwanjareul kanhosaka jjochneunda<br>patient(acc) nurse(nom) chase(pres ind)	20. 환자는 쫓나요 ?<br>hwanjaneun jjochnayo?<br>patient(top) chase	N

21a. 소년이 선생님에게 책을 준다.<br>sonyeoni seonsaengnimege chaekeul junda<br>boy(nom) teacher(dat) book(acc) give(pres ind)	**21b.선생님에게 소년이 책을 준다.**<br>**seonsaengnimege sonyeoni chaekeul junda**<br>**teacher(dat) boy(nom) book(acc) give(pres ind)**	**21. 선생님은 책을 주나요?**<br>**seonsaengnimeul chaekeul junayo?**<br>**teacher(top) book(acc) give(pres ind)**	**N**
22a. 간호사가 학생에게 연필을 준다.<br>kanhosaka haksaengege yeonpireul junda<br>nurse(nom) student(dat) pencil(acc) give(pres ind)	22b. 학생에게 간호사가 연필을 준다.<br>haksaengege kanhosaka yeonpireul junda<br>student(dat) nurse(nom) pencil(acc) give(pres ind)	22. 간호사는 연필을 주나요?<br>kanhosaneun yeonpireul junayo?<br>Nurse(top) pencil(acc) give(pres ind)	Y
23a. 여자가 아이에게 사탕을 준다.<br>yeojaka aiege satangeul junda<br>woman(nom) child(dat) candy(acc) give(pres ind)	23b. 아이에게 여자가 사탕을 준다.<br>aiege yeojaka satangeul junda<br>child(dat) woman(nom) candy(acc) give(pres ind)	23. 아이는 사탕을 주나요?<br>aineun satangeul junayo<br>child(top) candy(acc) give(pres ind)	N
24a. 의사가 환자에게 종이를 준다.<br>euisaka hwanjaege jongireul junda<br>doctor(nom) patient(dat) paper(acc) give(pres ind)	24b. 환자에게 의사가 종이를 준다.<br>hwanjaege euisaka jongireul jeunda<br>patient(dat) doctor(nom) paper(acc) give(pres ind)	24. 의사는 종이를 주나요?<br>euisaneun jongireul junayo ?<br>doctor(top) paper(accà give(pres ind)	Y
25a. 선생님이 간호사에게 모자를 전달한다.<br>seonsaengnimi kanhosaege mojareul joendalhanda<br>teacher(nom) nurse(dat) hat(acc) hand to(pres ind)	25b. 간호사에게 선생님이 모자를 전달한다.<br>kanhosaege seonsaengnimi mojareul jundalhanda<br>nurse(dat) teacher(nom) hat(acc) hand to(pres ind)	25. 간호사는 모자를 전달하나요?<br>kanhosaneun(top) mojareul(acc)<br>nurse(top) hat(acc) hand to(pres ind)	N
26a. 의사가 아이에게 종이를 전달한다.<br>euisaka aiege jongireul joendalhanda<br>doctor(nom) child(dat) paper(acc) hand to(pres ind)	26b. 아이에게 의사가 종이를 전달한다.<br>aiege euisaka jongireul joendalhanda<br>child(dat) doctor(nom) paper(acc) hand to(pres ind)	26. 의사는 종이를 전달하나요?<br>euisaneun jongireul joendalhanayo ?<br>doctor(top) paper hand to(pres ind) ?	Y
27a. 소녀가 남자에게 가방을 전달한다.<br>sonyeoka namjaege kabangeul joendalhanda<br>girl(nom) man(dat) bag(acc) hand to(pres ind)	27b. 남자에게 소녀가 가방을 전달한다.<br>namjaege sonyeoka kabangeul joendalhanda<br>man(dat) girl(nom) bag(acc) hand to(pres ind)	27. 남자는 가방을 전달하나요?<br>namjaneun kabangeul joendalhanayo<br>man(top) bag(acc) ghad to (pres ind)?	N
28a. 아이가 여자에게 우산을 전달한다.<br>aika yeojaege usaneul joendalhanda<br>child(nom) woman(dat) umbrella(acc) hand to(pres ind)	28b. 여자에게 아이가 우산을 전달한다.<br>yeojaege aika usaneul joendalhanda<br>child(nom) woman(dat) umbrella(acc) hand to(pres	28. 여자는 우산을 전달하나요?<br>yeojaneun usaneul joendalhanayo?<br>woman(top) umbrella(acc) hand to (pres	Y
29a. 학생이 간호사에게 책을 판다.<br>haksaengi kanhosaege chaekeul panda<br>student(nom) nurse(dat) book(acc) sell(pres ind)	29b. 간호사에게 학생이 책을 판다.<br>kanhosaege haksaengi chaekeul panda<br>nurse(dat) student(nom) book(acc) sell (pres ind)	29. 학생은 책을 파나요?<br>haksaengeun(top) chaekeul panayo?<br>student(top) book(acc) sell(pres ind)	Y
30a. 요리사가 남자에게 술을 판다.<br>yorisaka namjaege suleul panda<br>chef(nom) man(dat) alcohol(acc) sell(pres ind)	30b. 남자에게 요리사가 술을 판다.<br>namjaege yorisaka suleul panda<br>man(dat) chef(nom) alcohol(acc) sell(pres ind)	30. 남자는 술을 파나요?<br>namjaneun suleul panayo?<br>man(top) alcohol(acc) sell(pres ind)	N
311a. 소녀가 여자에게 꽃을 판다.<br>sonyeoka yeojaege kkocheul panda<br>girl(nom) woman(dat) flower(acc) sell(pres ind)	31b. 여자에게 소녀가 꽃을 판다.<br>yeojaege sonyeoka kkocheul panda<br>woman(dat) girl(nom) flower(acc) sell(pres ind)	31 . 여자는 꽃을 파나요?<br>yeojaneun kkocheul panayo?<br>woman(top) flower(acc) sell(pres ind)	N

32a. 어른이 소년에게 신문을 판다.<br>eoreuni sonyeonege shinmuneul panda<br>adult(nom) boy(dat) newspaper(acc) sell(pres ind)	32b. 소년에게 어른이 신문을 판다.<br>sonyeonege eoreuni shinmuneul panda<br>boy(dat) adult(nom) newspaper(acc) sell(pres ind)	32 . 어른은 신문을 파나요?<br>oereunen shinmeuneul panayo ?<br>adult(top) newspaper(acc) sell (pres ind)	Y
33a. 여자가 소녀에게 인사한다.<br>yeojaka sonyeoege insahanda<br>woman(nom) girl(dat) greet(pres ind)	33b. 소녀에게 여자가 인사한다.<br>sonyeoege yeojaka insahanda<br>girl(dat) woman(nom) greet(pres ind)	33. 소녀는 인사하나요?<br>sonyeoneun insahanayo ?<br>girl(top) greet(pres ind) ?	N
34a. 선생님이 소년에게 인사한다.<br>seonsaengnimi sonyeonege insahanda<br>teacher(nom) boy(dat) greet(pres ind)	34b. 소년에게 선생님이 인사한다.<br>sonyeonege seonsaengnimi insahanda<br>boy(dat) teacher(nom) greet(pres ind)	34. 선생님은 인사하나요?<br>seonsaengnimeun insahanayo ?<br>Teacher(top) greet(pres ind) ?	Y
35a. 환자가 의사에게 인사한다.<br>hwanjaka euisaege insahanda<br>patient(nom) doctor(dat) greet(pres ind)	35b. 의사에게 환자가 인사한다.<br>euisaege hwanjaka insahanda<br>doctor(dat) patient(nom) greet(pres ind)	35. 환자는 인사하나요?<br>hwanjaneun insahanayo ?<br>patient(top) greet(pres ind) ?	Y
36a. 남자가 요리사에게 인사한다.<br>namjaka yorisaege insahanda<br>man(nom) chef(dat) greet(pres ind)	36b. 요리사에게 남자가 인사한다.<br>yorisaege namjaka insahanda<br>chef(dat) man(nom) greet(pres ind)	36. 요리사는 인사하나요?<br>yorisaneun insahanayo ?<br>chef(top) greet(pres ind) ?	N
37a. 아이가 의사에게 이야기한다.<br>aika euisaege iyagihanda<br>child(nom) doctor(dat) say hello	37a. 의사에게 아이가 이야기한다.<br>euisaege aika iyagihanda<br>doctor(dat) child(nom) say hello(pres ind)	37. 아이는 이야기하나요?<br>Aineun iyagihanayo?<br>Child(top) sayhello (pres ind)?	Y
38a. 소년이 어른에게 이야기한다.<br>sonyeoni eoreunege iyagihanda<br>boy(nom) adult(dat) say hello(pres ind)	38b. 어른에게 소년이 이야기한다.<br>eoreunege sonyeoni iyagihanda<br>adult(dat) boy(nom) say hello(pres ind)	38. 소년은 이야기하나요?<br>Sonyeoneun iyagihanayo ?<br>Boy(top) say hello(pres ind) ?	Y
39a. 간호사가 선생님에게 이야기한다.<br>kanhosaka seonsaengnimege iyagihanda<br>nurse(nom) teacher(dat) say hello(pres ind)	39b. 선생님에게 간호사가 이야기한다<br>seonsaengnimege kanhosaka iyagihanda<br>teacher(dat) nurse(nom) say hello(pres ind)	39. 선생님은 이야기하나요?<br>Sansaengnimeul iyagihanayo ?<br>Teacher(top) say hello(pres ind) ?	N
40a. 남자가 소녀에게 이야기한다.<br>namjaka sonyeoege iyagihanda<br>man(nom) girl(dat say hello(pres ind)	40b. 소녀에게 남자가 이야기한다.<br>sonyeoege namjaka iyagihanda<br>man(nom) girl(dat say hello(pres ind)	40. 소녀는 이야기하나요<br>Sonyeoneun iyagihanayo ?<br>Girl(top) say hello(pres ind) ?	N
